# Honeydew Honey as a Reservoir of Bacteria with Antibacterial and Probiotic Properties

**DOI:** 10.3390/antibiotics13090855

**Published:** 2024-09-06

**Authors:** Dorota Grabek-Lejko, Mariusz Worek

**Affiliations:** 1 Department of Bioenergetics, Food Analysis and Microbiology, Institute of Food Technology and Nutrition, University of Rzeszow, Zelwerowicza 4 Street, 35-601 Rzeszow, Poland; 2 Department of Microbiology, Institute of Medical Sciences, University of Rzeszow, Kopisto 2a Avenue, 35-959 Rzeszow, Poland; mworek@ur.edu.pl

**Keywords:** honey, bacteria, antibacterial potential, MALDI-TOF-MS, foodborne pathogens, probiotics, *Bacillus* spp.

## Abstract

The purpose of this study was to isolate, identify, and evaluate the antibacterial and probiotic potential of bacteria from honeydew honey collected in Poland. Isolates (189 colonies from 10 honey samples) were evaluated for their antimicrobial activity against *Staphylococcus aureus*, *Bacillus cereus*, *Escherichia coli,* and *Yersinia enterocolitica*, and then identified by MALDI-TOF-MS. The isolates with the greatest antimicrobial properties were screened for their probiotic potential. The total number of bacteria isolated from honey did not exceed the value of 2.5 × 10^2^ CFU/mL. The *Bacillus pumilus*/*altitudinis*, *B. licheniformis*, and *Bacillus cereus* groups were the dominant identified bacteria. Almost 16% of the isolates expressed antibacterial potential against three pathogenic bacteria, over 20% against two, while almost 34% of the isolates did not inhibit any. The survival rate of the isolates under gastrointestinal tract conditions was higher after 4 h of exposure to bile salts (>60% survival rate for 66.66% of the isolates), while at pH 2.0, it was lower (>50% survival rate for 44% of the isolates). The most resistant isolate *B. pumilus*/*altitudinis* survived at a rate of 77% at low pH and 108% with bile salts. These results confirmed that honeydew honey is a promising reservoir of bacteria that produces metabolites with antimicrobial and probiotic potential.

## 1. Introduction

Due to the growing problem of antibiotic resistance [1,2] and the important health-promoting properties of probiotics [3,4,5,6], looking for new antibiotics and probiotics from various environments, including food products, like honey, seems to be a current research problem.

Honey is a natural sweet substance produced by *Apis mellifera*, which has been known and used for ages in traditional medicine due to its antibacterial, antiviral, antioxidant, anti-inflammatory, anticarcinogenic, and antimutagenic properties [7,8,9,10]. Honey bioactivity varies, and in general, dark honeys possess greater biological properties than light honeys. What is more, one of the most valuable dark honey varieties is honeydew honey [11,12]. Honeydew honey is produced by bees from excretions of plant-sucking insects or secretions of plants and possesses different properties to blossom honey produced from the flower nectar [12]. Honeydew honey is mainly produced from coniferous trees, such as fir and spruce, and can also be produced from leafy trees such as oak and lime [11]. The south-eastern region of Poland (Podkarpackie Voivodeship), with long traditions in beekeeping, is an ecologically clean region, rich in coniferous forests, with fir domination, and is a perfect place where honeydew honey can be collected. Podkarpackie honeydew honey was registered by the European Commission as a Protected Designation of Origin in 2010 [13] and possesses a higher than normative content of simple sugars and moderate acidity with a dark color, which comes from the type of fir grown in this area [12]. The higher content of bioactive compounds such as phenolics, proteins, amino acids, enzymes, and minerals in honeydew honey, compared to blossom honey, influences its higher biological properties. Moreover, the botanical and geographical origin of plants can influence the microbial composition of honey [10,11,12]. For this reason, Podkarpackie honeydew honey was used as a source of bacteria with potential antimicrobial and probiotic properties.

The main species in the honey microbiota are bacterial species belonging to the genus *Bacillus* and *Lactobacillus*. These bacteria are also well-known probiotics [10,14,15,16,17]. Probiotics are living microorganisms that when administered in adequate amounts could provide health benefits to the host (FAO/WHO, 2006) [18]. The beneficial effect corresponds to improving the balance and restoration of the gut microbiota, promoting immunomodulation and anticancer effects, managing lactose intolerance, reducing blood pressure and cholesterol content, lowering allergic symptoms, and preventing urogenital symptoms [17].

The most common probiotic bacteria belong to the genera *Lactobacillus* and *Bifidobacterium*. These probiotics have some drawbacks such as slow growth, a short shelf life in food products, and high temperature sensitivity. Moreover, these microorganisms are strict anaerobes or microaerophiles, so their production requires complicated and more expensive procedures. Researchers are looking for other potential probiotic strains which can be more resistant to bile salts, be tolerant to low pH, have a long shelf life, result from easier production methods, and be more resistant to food processing (like cooking) and storage conditions [5,17,19].

Recently, *Bacillus* spp. have gained much attention as potential probiotics. *Bacillus* is closely related to *Lactobacillus* but, due to spore formation, is very resistant to stress conditions, such as high temperature, harsh conditions in the gastrointestinal tract, etc., compared to other probiotics [20]. Using *Bacillus* as a probiotic offers numerous potential benefits for developing a wider range of food products as probiotic carriers [5].

The purpose of this study was to identify and analyze the antibacterial potential of bacteria isolated from honeydew honey samples collected in south-eastern Poland (Podkarpackie Voivodeship) against the food pathogens chosen. Another purpose of this study was to analyze whether bacteria with excellent antimicrobial properties can also be used as probiotics. For this purpose, tolerance to low pH and a high content of bile salts and antibiotic susceptibility were examined.

To our knowledge, this is the first study in which bacteria isolated from Podkarpackie honeydew honey were identified and screened for their possible probiotic and antimicrobial properties.

## 2. Results

### 2.1. Total Content of Bacteria

**Figure 1:**
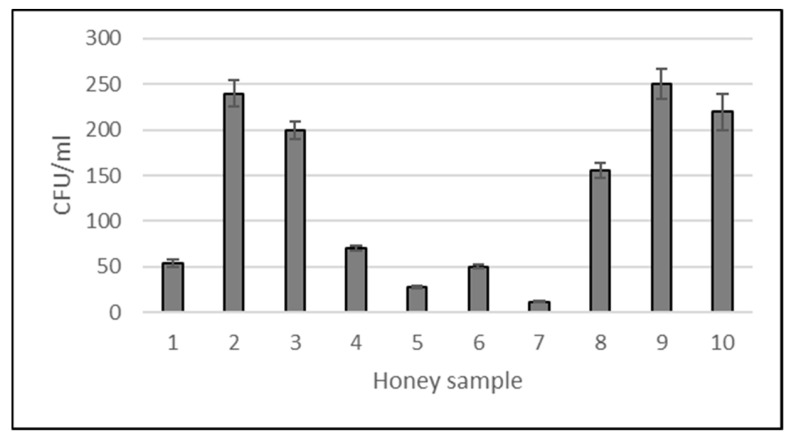
Total number of bacteria in honeydew honey samples (CFU/mL). Mean ± SD of three replications.

The total number of bacteria isolated from honeydew honey samples varied from 1.2 × 10^1^ CFU/mL to 2.5 × 10^2^ CFU/mL (only aerobic or facultative aerobic bacteria were taken into account). The lowest level of bacteria was observed for sample no. 7 and the highest for honey no. 9 (Figure 1). Generally, honey samples were contaminated by bacteria at a low level.

### 2.2. Antibacterial Potential of Bacteria Isolated from Honey

**Figure 2:**
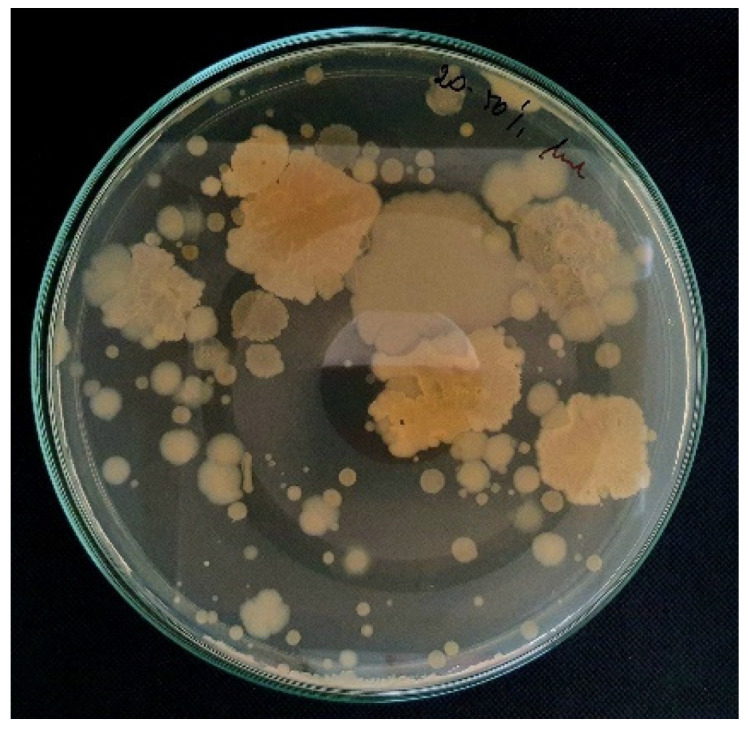
Microorganisms isolated from honeydew honey samples (grown in TSA medium).

**Figure 3:**
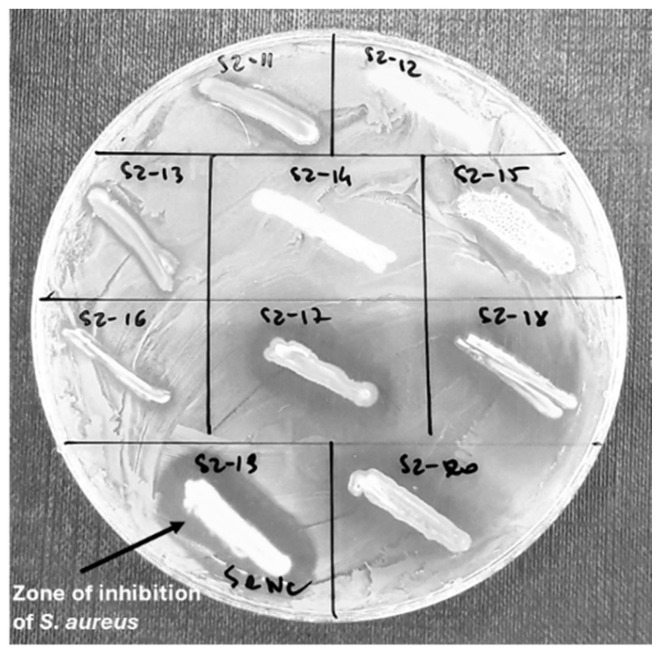
The antibacterial potential of honey-isolated microorganisms (a representative plate with visible inhibition zones of *S. aureus* around the growth of some honey isolates).

**Figure 4:**
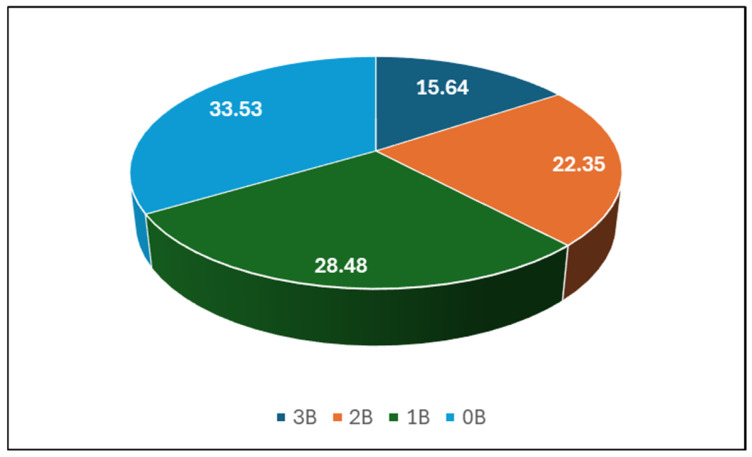
Percentage of bacterial isolates with resistance to three (3B), two (2B), one (1B), and zero (0B) reference bacterial strains.

Finally, 189 isolates (Figure 2) were selected for their antibacterial potential (Figure 3). Among these isolates, 15.64% of microorganisms expressed antibacterial potential against three reference bacteria, more than 20% of isolates inhibited the growth of two pathogenic bacteria, while 28.48% expressed antibacterial potential against only one bacterial strain. Almost 34% of the microorganisms isolated from the honeydew honey samples did not inhibit any of the reference bacterial strains (Figure 4). In nine honeydew honey samples, at least one isolate exhibited antibacterial potential against three pathogenic bacteria, while in one sample, at least one isolate exhibited antibacterial potential against at least one pathogenic strain, which was also previously observed by Pajor et al. [14] for honey collected from northern Poland. Higher antibacterial potential was observed against Gram-positive bacteria (*S.aureus* and *B. cereus*), with 47.09% and 45.5% of isolates inhibiting the growth of these bacteria; however, stronger inhibition was observed against *S. aureus*. Only 16.4% of the isolates exhibited antibacterial potential against *E. coli* and 4.76% against *Y. enterocolitica* (Table 1).

### 2.3. The Identification of Bacteria Isolated from Honeydew Honey

**Figure 5:**
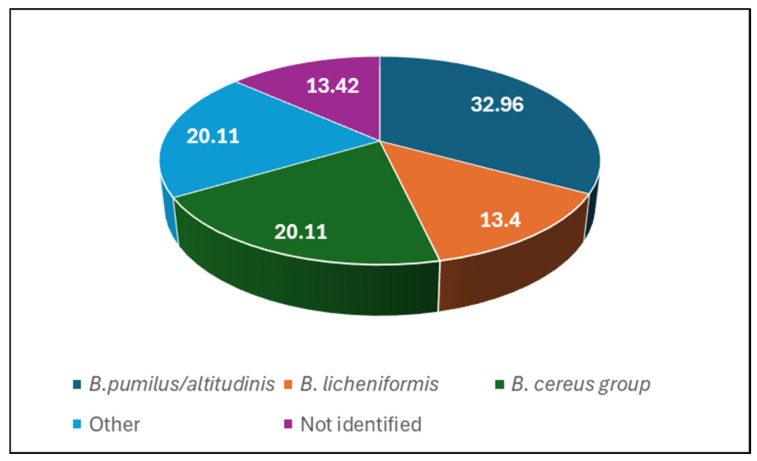
The percentage of bacterial species identified in honeydew honey samples. Other means bacteria represented by a small number of isolates.

The MALDI-TOF-MS method revealed that all isolates were classified as Gram-positive bacteria that belong mainly to the genus *Bacillus*. The most frequently identified was *B. pumilus*/*altitudinis* (32.96%), followed by the *B. cereus* group (20.11%) and *B. licheniformis* (13.4%) (Figure 5). Among other bacteria isolated in small numbers, we can mention the following: the genus *Paenibacillus* (*P. thiaminolyticus*, *P. pabuli*, *P. amylolyticus*), belonging to the genus *Bacillus* (*B. subtilis/amyloliquefaciens/vallismortis*, *B. megaterium*, *B. simplex*, *B. galactosidilyticus*, *B. circulans*, *B. clausii*, *B. flexus*), and the genera *Lysinibacillus fusiformis* and *Virgibacillus proomi*. Among non-spore-forming bacteria, *Staphylococcus epidermidis* was identified. Thirteen percent of isolated samples were not identified, probably due to the lack of reference spectra in the apparatus database.

Eighteen isolates of *Bacillus* spp. with the strongest inhibition of three pathogenic bacteria were chosen for further analysis (fourteen isolates of *B. pumilus*/*altitudinis*, and two isolates of the *B. subtilis* and *B. cereus* groups).

### 2.4. Probiotic Properties

Since low pH in the stomach and bile salts in the intestine are the first biological barriers that probiotic bacteria must overcome after ingestion to reach their place of action, acid and bile resistance are the most essential factors for the viability and growth of probiotic strains during their passage to the gastrointestinal tract [21,22]. For this reason, we decided to determine the survival rate of the *Bacillus* isolates after 4 h of storage at low pH (2.0) and in the presence of 0.3% bile salts.

#### 2.4.1. Resistance to Bile Salts and Low pH

The survival rate of the isolates under gastrointestinal tract conditions is presented in Table 2. After exposure to low pH (2.0), the survival rate of the bacterial isolates was different. Some isolates did not survive at this pH after 4 h of exposure (five strains). Two of these strains belonged to the *B. cereus* group (no. 6 and 12) and three to *B. pumilus*/*altitudinis* (no. 4, 8, and 9). However, a survival rate of more than 50% was observed for eight strains (44.44%). Higher tolerance was observed for bile salts. Only three isolates did not survive after 4 h of exposure to bile salts. Among these strains, two belonged to the *B. cereus* group (no. 6 and 12) and one was *B. pumilus*/*altitudinis*. The survival rate of 12 isolates (66.66%) was higher than 60%. The most resistant strains to gastrointestinal tract conditions were strains 5, 16, and 17. The survival rates of two strains of *B. pumilus*/*altitudinis* isolated from honeydew honey in the presence of 0.3% bile salts ranged from 96.59 to 107.97%, while at low pH, these strains survived at the level of 60.67–76.66%.

#### 2.4.2. Antibiotic Sensitivity Test

Regarding the evaluation of the antibiotic resistance of *Bacillus* isolates (Table 3), all were sensitive to gentamicin and erythromycin, and sensitive or moderately sensitive to doxycycline, clindamycin, chloramphenicol, and streptomycin (with one exception of the resistant strain). Two isolates were resistant to ampicillin and three to tetracycline. The largest number of isolates were resistant to amoxicillin and cefoxitin, 13/18 and 6/18, respectively. In summary, *Bacillus* isolates were mainly sensitive to the antibiotics analyzed. Only three isolates were resistant to three antibiotics (no. 6, 12, and 13). The most resistant isolates were *B. cereus* (no. 6 and 12) (no inhibition zone observed against two and three antibiotics).

## 3. Discussion

The properties of honey, such as low pH, high osmotic pressure, and the presence of some antimicrobial agents, such as hydrogen peroxide, bee defensin-1, and phytochemicals, successfully inhibit the growth and reproduction of bacteria in honey [8,9,14]. The low level of microbiological contamination was confirmed in our investigation and was previously also observed by other authors, with the total bacteria content below 10^3^ CFU/mL [23,24,25,26,27].

Today, the growing resistance of bacteria to antibiotics is one of the most serious public health issues [1]. To address these problems, scientists are constantly looking for new antibiotics from various environments, including food products [2]. The bacteria isolated from honeydew honey were analyzed for their antimicrobial properties against food pathogens. The strongest antimicrobial potential was observed against Gram-positive bacteria, and weaker potential was seen against Gram-negative ones. The stronger resistance of Gram-negative bacteria may be related to the structure of their cell wall. Gram-negative bacteria contain an outer membrane with a hydrophilic structure (rich in lipopolysaccharides) and a unique periplasmic space, which is not present in Gram-positive bacteria [28]. All honey samples contained bacteria producing anti-Gram-positive bacterial agents. Similarly, a higher antibacterial potential against Gram-positive bacteria (*S. aureus*, *Listeria monocytogenes*) than against Gram-negative bacteria (*E. coli* and *Pseudomonas aeruginosa*) was described for bacteria isolated from honey collected in northern Poland [14]. The isolates without antibacterial properties belonged mainly to *B. licheniformis*, *B. clausii*, *B. subtilis/amyloliquefaciens/vallismortis*, *B. circulans*, *B. simplex*, *L. fusiformis*, *Paenibacillus* sp., and *S. epidermidis*. Eight isolates without antibacterial potential were not identified. These results suggest that *B. pumilus*/*altitudinis*, present in Podkarpacki honeydew honey, is the most prospective bacteria due to having the strongest antimicrobial potential against food pathogens.

MALDI-TOF-MS was used for the identification of bacterial isolates. The bacteria most frequently present belonged to *Bacillus* spp. (*B. pumilus*/*altitudinis*, *B. licheniformis*, and *B. cereus* groups). *Bacillus* spp. are widely spread in fermented foods, the human gut, and the environment, especially in soil, air, and plants, which may justify the fact that they are frequently present in honey, while the botanical and geographical origin of plants can influence the microbial composition of honey [10]. Similarly, Tsadila et al. [29] detected that the most frequently identified species of Greek honey were *Bacillus safensis* (unidentified in this study), then *B. pumilus* (16.1%), *B. subtilis* (12.9%), and *B. cereus* (6.5%). Furthermore, *B. pumilus* (34%) and *B. altitudinis* (33%) dominated in Malaysian stingless bee honey [24]. In studies by López et al. [30] and Alippi et al. [31], the aerobic spore-forming bacteria belonging to *Bacillus* spp. and related genera (*Lysinibacillus*, *Paenibacillus*) have been identified as the most frequently present in honey. Similarly, in Argentinian honey, the most frequently identified bacteria were *B. cereus* (26%), *B. laterosporus* (26%) (not identified in honeydew honey samples), and *B. pumilus* (13%) [23]. The most representative bacteria isolated from Italian honey belong to the *Bacilli* class. In addition to these microorganisms, bacteria producing lactic acid and food contaminants such as *Acinetobacter*, *Propionibacterium*, and *Pseudomonas* were identified [32]. In addition to spore-forming bacteria, only *S. epidermidis* was identified in our study, with no other bacterial pathogens. Similarly, Iurlina and Fritz [23] did not find pathogenic bacteria in Argentinian honey. However, they detected *Paenibacillus larvae* subspp. larvae, which are very dangerous bacteria for bees and are responsible for American foulbrood disease. We did not detect this bacterium in our honey samples, but other representatives of the *Paenibacillus* genus were present (*P. thiaminolyticus*, *P. pabuli*, and *P. amylolyticus*). The isolated bacteria from honey samples collected in north Poland were the same as in our study [14]. The authors identified *B. pumilus*, *B. licheniformis*, *B. altitudinis*, *B. megaterium, Paenibacillus* sp., and *Lysinibacillus* sp. They also isolated strains of *Staphylococcus* sp.

Taking into account that the MALDI-TOF MS technique is successfully used for the identification of clinical strains and is still limited in the identification of non-pathogenic species from different matrices [33], it can be assumed that the unidentified isolates do not belong to pathogenic microorganisms.

Due to the numerous human health benefits of probiotic ingestion and the growing attention on *Bacillus* spp. as potential probiotics caused by the production of resistant cells [19,34], we also decided to investigate the probiotic potential of isolates with the strongest antibacterial properties. *B. pumilus*/*altitudinis*, *B. licheniformis, B. cereus*, and *B. subtilis* are known as probiotics and are present in many commercial probiotics [17].

Due to the high level of survival rates under gastrointestinal tract conditions, few strains of *B. pumilus*/*altitudinis* isolated from honeydew honey can compete with LAB (lactic acid bacteria). For example, the survival rates of LAB, isolates from Ethiopian traditional fermented foods and beverages, ranged from 57.56 to 99.20% in the presence of 0.3% bile salt content, and from 34.92 to 98.82% at low pH [35].

The growing global impact of antibiotic resistance is a huge problem nowadays. The large number of probiotic bacteria in foodstuffs has excellent chances of spreading resistant determinants, especially when sharing residence with intestinal microflora and opportunistic pathogens in the host’s intestinal tract. Probiotic bacteria may harbor antibiotic resistance genes themselves and may be involved in their dangerous transfer to other microorganisms, including pathogenic bacteria [3]. For this purpose, the susceptibility to antibiotics of isolated *Bacillus* strains was tested against two groups of antibiotics with different mechanisms of action. One group contained antibiotics such as streptomycin, ampicillin, amoxicillin, and cefoxitin, which are cell wall inhibitors, while chloramphenicol, erythromycin, tetracycline, gentamicin, doxycycline, and clindamycin are protein synthesis inhibitors. It was shown that most isolates were sensitive to different antibiotics, and only a few isolates were totally resistant to a few antibiotics; some were resistant to cefoxitin and amoxicillin (inhibition zones ≤ 15 mm). According to published data, bacilli are often resistant to antibiotics of the penicillin group (β-lactam antibiotics), including cefoxitin and amoxicillin, which suggests that these isolates possess beta-lactamases, enzymes aimed at combating β-lactam antibiotics [36,37]. However, due to the lack of a reliable interpretation document for the disc diffusion method for *Bacillus* spp., some authors [6] assumed that inhibition zones lower than 12 mm were considered resistant to antibiotics. Based on this interpretation, all *B. pumilus*/*altitudinis* isolates were susceptible to antibiotics, and we can speculate that these isolates (with a high survival rate in gastrointestinal tract conditions and susceptibility to antibiotics) should be considered safe probiotics for humans and animals.

In summary, the antibacterial potential of *Bacillus* strains isolated from honeydew honey collected in Podkarpackie, Poland, has been proven. Moreover, for the first time, preliminary results of the probiotic properties of *Bacillus* strains have also been confirmed. However, additional analyses should be performed, such as assessing bacterial capacity for aggregation, hydrophobicity, hemolysis, and lecitinase activity, which can be a health risk for the host. Furthermore, the ability to transfer genes related to antibiotic resistance, the production of enterotoxins and biogenic amines, and cytotoxicity against normal cells must be investigated to ensure the safety of *Bacillus* strains isolated from honeydew honey. These results confirm that Podkarpacki honeydew honey is a promising reservoir of bacteria, especially *B. pumilus*/*altitudinis*, that produce metabolites with antimicrobial and probiotic potential and, due to their high resistance to harsh conditions and antibiotic susceptibility, can be considered safe probiotics and possible additives to food products.

## 4. Materials and Methods

### 4.1. Microbial Content of Honey

Ten honeydew honey samples were collected directly from beekeepers working in the south-eastern part of Poland (Podkarpackie Voivodeship) in 2021 and 2022. The samples were stored at room temperature, in the dark, until analysis. The total number of mesophilic bacteria was determined by spreading 1 mL of 50% honey solution in water (*m*/*v*) on the surface of NA (Nutrient Agar, Biomaxima, Lublin, Poland) plates (20 cm in diameter). The plates were incubated for 24 h at 37 °C; then, colonies were calculated and the results were presented as CFU/mL of honey (only aerobic bacteria were analyzed) [14].

### 4.2. Antibacterial Potential of Bacteria Isolated from Honey

Twenty randomly selected bacterial colonies (from each honey sample) with different morphological appearances were selected for further analysis and re-cultured on TSA plates (total isolates—186). The antibacterial properties of the selected bacteria were analyzed against *S. aureus* ATCC 6538P, *Bacillus cereus* PCM 482, *Escherichia coli* ATCC 8739, and *Yersinia enterocolitica* PCM 2080. For this purpose, the bacteria were incubated overnight at 37 °C on TSA medium. The suspension of each reference strain tested was then prepared with the final optical density OD = 0.132. Bacterial solutions (0.1 mL) were spread on the MHA plates (Mueller–Hinton Agar, Biomaxima, Lublin, Poland) and dried in the laminar chamber; then, overnight cultures (16 h of incubation, 37 °C) of honey-isolated microbial colonies were transferred by a sterile loop (in the form of a short line—1–1.5 cm) onto the MHA plates with the appropriate reference strain. The plates were incubated for 24 h at 37 °C. Then, halo zones of inhibition of reference bacteria were observed [14].

### 4.3. Identification of Bacteria by MALDI-TOF MS

The strains were identified using the MALDI-TOF MS technique and the VITEK MS system (bioMérieux, Warsaw, Poland). For this purpose, the chosen colonies were incubated according to the producer’s instructions (TSA medium, 16 h of incubation at 37 °C). The bacterial biomass was then spotted on the target slide and dried in air at room temperature. The sample spots were then covered with 1 µL of VITEK MS-CHCA matrix and air-dried until the matrix and the sample were co-crystallized. The target slide was loaded onto the VITEK MS system to acquire the mass spectra of the whole bacterial cell protein. The obtained mass spectra were compared with the known mass spectra contained in the database, and a confidence score was given (99.9%) according to the degree to which the acquired spectra were close to those contained in the database. Each target plate comprised a single spot of pure matrix solution, used as a negative control, and a spot of Bacterial Test Standard (*E. coli* ATCC 8739), used for calibration.

### 4.4. Probiotic Properties

#### 4.4.1. Resistance to Bile Salts and Low pH

Resistance to bile salts and tolerance to acidic conditions were determined according to Amin et al. [24], with slight modifications. For this purpose, the chosen isolates were incubated overnight in TSA medium at 37 °C. The tolerance of isolates to bile salts (Sigma-Aldrich, Burlington, MA, USA) was evaluated by inoculating 30 µL of bacterial suspension in nutrient broth (Biomaxima, Poland) (3 mL) comprising 0.3% of salts, while for pH resistance, the same volume of bacterial suspension was added to the nutrient broth adjusted to pH = 2.0 (with HCl). The final bacterial concentration was around 1 × 10^6^ CFU/mL. As a control, pure nutrient broth was used. The samples were then incubated for 4 h at 37 °C. The samples at 0 h and 4 h were serially diluted, spread on TSA plates, and incubated at 37 °C for 24 h. Cell viability was assessed by determining the CFU/mL (colony forming units), and the survival rate (SR) was calculated according to the equation below:

SR = C4/C0 × 100%,

where C4 (log CFU/mL) is the number of total viable colonies of the selected isolates after treatment (t = 4 h), and C0—(log CFU/mL)—is the number of total viable colonies of the selected isolates before treatment (t = 0 h).

#### 4.4.2. Antibiotic Sensitivity Test

The disc diffusion method was used for the measurement of antibiotic sensitivity to evaluate the resistance of the isolates. A bacterial suspension from the fresh, night culture on TSA medium was adjusted to a 0.5 McFarland standard and spread (0.1 mL) on a Mueller–Hinton agar plate. Then, antibiotic discs (S10—streptomycin 10 μg, FOX30—cefoxitin 30 μg, AX25—amoxicillin 25 μg, AM10—ampicillin 10 μg, TE30—tetracycline 30 μg, CN10—gentamicin 10 μg, E15—erythromycin 15 μg, C30—chloramphenicol 30 μg, DO30—doxycycline 30 μg, DA2 and clindamycin 2 μg) purchased from Biomaxima, Lublin, Poland, were placed on the plates. The plates were incubated at 37 °C for 24 h, and then, the diameters of the inhibition zones were measured. The results were expressed in terms of resistance (≤15 mm), moderate susceptibility (16–20 mm), or susceptibility (≥21 mm) [4].

## 5. Conclusions

In conclusion, some species of *Bacillus* spp. isolated from Polish honeydew honey (with dominant *B. pumilus*/*altitudinis*) showed good probiotic properties, such as resistance to acid and bile salts and antimicrobial activity against foodborne pathogens. These results suggest that Podkarpackie honeydew honey from south-eastern Poland can be used as a good source of microorganisms with antibacterial potential, which can be useful for the prevention or treatment of foodborne infections, and as probiotic supplements in different food products. However, additional studies on probiotic properties, like an evaluation of their stability through different food processes and storage conditions, must be conducted in the future.

## Figures and Tables

**Table 1:** Number of bacterial isolates with antimicrobial potential.

Honey Sample	*S. aureus*	*B. cereus*	*E. coli*	*Y. enterocolitica*
1	15	11	5	0
2	11	18	3	3
3	12	12	6	0
4	8	2	4	2
5	8	5	3	2
6	4	1	0	0
7	11	7	5	1
8	6	14	4	1
9	5	6	1	0
10	9	10	0	0
Total	89	86	31	9
%	47.09	45.5	16.4	4.76

**Table 2:** Survival of *Bacillus* isolates in gastrointestinal tract conditions.

Isolates	Survival Rates, %	
	Acid Tolerance (pH = 2.0)	Bile Tolerance (0.3%)
1	59.90	79.06
2	47.45	81.06
3	49.50	85.77
4	0.00	42.01
5	66.95	85.99
6	0.00	0.00
7	40.44	74.09
8	0.00	50.35
9	0.00	0.00
10	37.93	44.87
11	36.36	62.47
12	0.00	0.00
13	58.49	70.98
14	52.26	63.64
15	63.46	78.05
16	76.66	107.97
17	60.67	96.59
18	54.42	72.32

Bacterial isolates: 1–5, 7–11, 14, and 16–18—*B. pumilus*/*altitudinis*; 6 and 12—*B. cereus* group; 13 and 15—*B. subtilis*.

**Table 3:** Antibiotic susceptibility of *Bacillus* isolates.

Isolate	Diameter of Inhibition Zone (mm)									
	S10	FOX30	AX25	AM10	TE30	CN10	E15	C30	DO30	DA2
1	23.3 ± 0.6 (S)	17.0 ± 1.0 (MS)	14.0 ± 1.5 (R)	32.3 ± 1.2 (S)	24.5 ± 1.0 (S)	28.3 ± 1.5 (S)	26.0 ± 1.0 (S)	25.3 ± 0.6 (S)	28.3 ± 1.5 (S)	26.3 ± 0.6 (S)
2	24.7 ± 0.6 (S)	16.3 ± 0.6 (MS)	16.0 ± 1.0 (MS)	32.7 ± 1.2 (S)	24.7 ± 0.6 (S)	28.7 ± 1.2 (S)	30.0 ± 0.0 (S)	24.0 ± 1.0 (S)	28.3 ± 1.4 (S)	23.3 ± 1.2 (S)
3	19.7 ± 0.6 (MS)	13.3 ± 0.6 (R)	14.0 ± 1.0 (R)	24.3 ± 0.6 (S)	22 ± 1.0 (S)	21.0 ± 1.0 (S)	23.3 ± 0.6 (S)	25.3 ± 1.2 (S)	27.3 ± 1.5 (S)	24.0 ± 1.0 (S)
4	27.0 ± 1.0 (S)	18.0 ± 1.0 (MS)	15.0 ± 1.0 (R)	30.3 ± 2.1 (S)	21.7 ± 0.6 (S)	30.7 ± 0.6 (S)	28.3 ± 1.5 (S)	25.0 ± 1.0 (S)	30.3 ± 1.5 (S)	27.3 ± 0.6 (S)
5	20.0 ± 0.0 (MS)	15.0 ± 2.0 (R)	17.7 ± 0.6 (MS)	23.3 ± 1.5 (S)	23.0 ± 2.0 (S)	24.3 ± 0.6 (S)	29.3 ± 1.5 (S)	19.7 ± 2.1 (MS)	26.0 ± 1.0 (S)	10.0 ± 0.0 (R)
6	25.3 ± 2.1 (S)	NI (R)	NI (R)	7.0 ± 2.0 (R)	25.7 ± 1.5 (S)	25.3 ± 0.6 (S)	30.0 ± 2.0 (S)	26.7 ± 0.6 (S)	22 ± 1.0 (S)	17.0 ± 1.0 (MS)
7	22 (S)	14.3 ± 1.5 (R)	11.0 ± 2.0 (R)	25.0 ± 2.0 (S)	26.3 ± 1.2 (S)	25.0 ± 1.0 (S)	28.7 ± 1.2 (S)	NI (R)	27.0 ± 2.0 (S)	19.7 ± 0.6 (MS)
8	16.0 ± 1.5 (MS)	22.3 ± 2.1 (S)	15.3 ± 0.6 (R)	27.3 ± 0.6 (S)	19.7 ± 0.6 (MS)	30.0 ± 2.0 (S)	30.3 ± 1.2 (S)	33.0 ± 2.0 (S)	24.0 ± 1.0 (S)	25.3 ± 0.6 (S)
9	20.0 ± 1.0 (MS)	15.7 ± 0.6 (R)	12.5 ± 0.5 (R)	28.3 ± 2.0 (S)	27.0 ± 1.0 (S)	29.0 ± 2.0 (S)	29.7 ± 0.6 (S)	25.3 ± 0.6 (S)	30.0 ± 2.0 (S)	25.3 ± 3.1 (S)
10	22.7 ± 1.2 (S)	19.0 ± 2.0 (MS)	20.3 ± 0.6 (MS)	31.3 ± 1.5 (S)	30.2 ± 1.3 (S)	23.3 ± 0.6 (S)	30.0 ± 2.0 (S)	26.7 ± 0.6 (S)	32.3 ± 1.2 (S)	30.3 ± 1.5 (S)
11	26.7 ± 1.5 (S)	16.0 ± 2.0 (MS)	15.3 ± 0.6 (R)	30.7 ± 1.5 (S)	27.0 ± 1.0 (S)	30.3 ± 2.1 (S)	32.7 ± 1.2 (S)	23.3 ± 0.6 (S)	30.0 ± 1.0 (S)	27.7 ± 1.2 (S)
12	18.0 ± 2.0 (MS)	NI (R)	NI (R)	NI (R)	25.7 ± 0.6 (S)	25.0 ± 1.0 (S)	32.3 ± 1.2 (S)	30.7 ± 0.6 (S)	25.3 ± 1.5 (S)	25.7 ± 0.6 (S)
13	14.0 ± 0.0 (R)	17.3 ± 0.6 (MS)	13.0 ± 2.0 (R)	26.7 ± 1.5 (S)	15.0 ± 1.0 (R)	30.7 ± 1.2 (S)	33.3 ± 0.6 (S)	32.0 ± 2.0 (S)	27.3 ± 1.6 (S)	23.7 ± 1.2 (S)
14	18.7 ± 0.6 (MS)	18.0 ± 2.0 (MS)	15.3 ± 0.6 (R)	31.0 ± 2.0 (S)	25.7 ± 0.6 (S)	27.0 ± 1.0 (S)	30.7 ± 1.2 (S)	24.0 ± 2.0 (S)	28.3 ± 0.6 (S)	22.0 ± 0.0 (S)
15	16.0 ± 1.0 (MS)	16.0 ± 1.0 (MS)	19.7 ± 1.5 (MS)	22.0 ± 2.0 (S)	13.0 ± 0.0 (R)	25.3 ± 0.6 (S)	30.0 ± 2.0 (S)	28.3 ± 1.5 (S)	16.0 ± 2.0 (MS)	27.7 ± 0.6 (S)
16	20.7 ± 1.5 (MS)	13.0 ± 2.0 (R)	9.0 ± 1.0 (R)	24.7 ± 0.6 (S)	20.0 ± 1.0 (MS)	25.0 ± 2.0 (S)	25.3 ± 0.6 (S)	24.0 ± 1.0 (S)	30.0 ± 2.0 (S)	19.0 ± 1.0 (MS)
17	17.0 ± 1.0 (MS)	16.3 ± 1.5 (MS)	14.0 ± 1.0 (R)	25.3 ± 0.6 (S)	12.0 ± 1.0 (R)	27.0 ± 1.0 (S)	33.3 ± 0.6 (S)	32.0 ± 2.0 (S)	18.3 ± 1.2 (MS)	25.3 ± 1.2 (S)
18	22.3 ± 2.1 (S)	17.0 ± 1.0 (MS)	16.0 ± 0.0 (MS)	28.3 ± 0.6 (S)	30.7 ± 0.6 (S)	25.3 ± 1.5 (S)	31.0 ± 2.0 (S)	28.7 ± 0.6 (S)	31.3 ± 1.2 (S)	24.0 ± 1.0 (S)
*B. c.*	20.0 ± 2.0 (MS)	10.0 ± 1.0 (R)	-	NI (R)	24.3 ± 0.6 (S)	22.0 ± 1.0 (S)	30.7 ± 1.2 (S)	22.0 ± 2.0 (S)	27.3 ± 0.6 (S)	21.0 ± 1.0 (S)

Mean ± SD expressing inhibition diameter data (mm) in three replications. S—sensitive, MS—moderately sensitive, R—resistant, NI—no inhibition zone was observed, and *B. c*.—*Bacillus cereus*. Antibiotics: S10—streptomycin, FOX30—cefoxitin, AX25—amoxicillin, AM10—ampicillin, TE30—tetracycline, CN10—gentamicin, E15—erytromycin, C30—chloramphenicol, DO30—doxycycline, and DA2—clindamycin. Bacterial isolates: 1–5, 7–11, 14, and 16–18—*B. pumilus*/*altitudinis*; 6 and 12—*B. cereus* group; 13 and 15—*B. subtilis*.

## Data Availability

The data are contained within the article.
